# Influence of Dentin Surface Roughness, Drying Time, and Primer Application on Self-adhesive Composite-Cement Bond Strength

**DOI:** 10.3290/j.jad.b2916387

**Published:** 2022-04-13

**Authors:** Sung-Ae Son, Bit-Na Kim, Jae-Hoon Kim, Deog-Gyu Seo, Jeong-Kil Park

**Affiliations:** a Associate Professor, Department of Conservative Dentistry, Dental and Life Science Institute, School of Dentistry, Pusan National University, Dental Research Institute, Yangsan, Republic of Korea. Experimental design, performed the experiments, wrote the manuscript.; b Graduate Student, Department of Conservative Dentistry, School of Dentistry, Pusan National University, Yangsan, Republic of Korea. Performed the experiments in partial fulfillment of requirements for a degree.; c Assistant Professor, Department of Dental Education, Dental and Life Science Institute, School of Dentistry, Pusan National University, Dental Research Institute, Yangsan, Republic of Korea. Consulted on and performed statistical evaluation.; d Professor, Department of Conservative Dentistry and Dental Research Institute, School of Dentistry, Seoul National University, Seoul, Republic of Korea. Proofread the manuscript, contributed substantially to discussion.; e Professor, Department of Conservative Dentistry, Dental and Life Science Institute, School of Dentistry, Pusan National University, Dental Research Institute, Yangsan, Republic of Korea. Experimental design, proofread the manuscript, contributed substantially to discussion.; * Contributed equally to this work.

**Keywords:** self-adhesive composite cement, self-adhesive composite-cement primer, smear layer, dentin drying time, microtensile bond strength, confocal laser scanning microscopy

## Abstract

**Purpose:**

To investigate the effect of roughness and drying time of dentin as well as the number of coats of a self-adhesive composite-cement primer on the bond strength of self-adhesive composite cement.

**Material and Methods:**

Sixty human teeth were prepared and assigned to 12 groups (n = 5), according to three experimental factors: 1) dentin surface roughness, rough or fine, as achieved by 250- and 600-grit silicon carbide papers, respectively; 2) dentin wetness based on air-drying time (5 or 10 s); and 3) the self-adhesive composite-cement primer applications (no-coat, 1-coat, and 2-coat). Composite resin blocks were made with hybrid composite resin (M1 GraceFil) and cemented with G-CEM ONE (both GC). Cement-dentin sticks (12) were prepared, and the microtensile bond strength (μTBS) test was performed. Failure modes were observed with a stereomicroscope (40X), and bonding interfaces were evaluated with confocal laser scanning microscopy (CLSM). Statistical analysis was performed using three-way ANOVA and Tukey’s post-hoc comparisons test (α = 0.05).

**Results:**

Dentin roughness (250-grit > 600-grit, p = 0.000), drying time (5-s drying > 10-s drying, p = 0.000), and primer application (no-coat < 1-coat = 2-coat, p = 0.000) had significant effects on bond strength. These factors also showed significant interactions with each other (p = 0.003). The highest μTBS (31.8 ± 3.1 MPa) was observed in the 1-coat/fine roughness/10-s drying group and the lowest μTBS (13.4 ± 2.7 MPa) in the no-coat/coarse roughness/5-s drying group. CLSM showed higher penetration of cement in the primer-coated groups compared to that in the no-coat groups.

**Conclusion:**

Bond strength between the self-adhesive composite cement and dentin was higher in the fine-roughness dentin group than in the coarse-roughness dentin group, and in the 5-s drying group compared to the 10-s drying group. Applying a primer to dentin improved bond strength of the self-adhesive composite cement.

Self-adhesive composite cement consists of functional acidic monomers that are self-etching during the initial stage of a chemical reaction.^4^ This type of composite cement is applied to the tooth substrate without any additional acid treatment or adhesive application. Owing to the simplicity of clinical application and the advantageous mechanical properties, self-adhesive composite cements have been widely used for bonded indirect restorations in recent years.^1,8^

Similar to self-etching adhesives, self-adhesive composite cement allows wetting of the tooth structure, due to its low pH and high hydrophilicity in the initial stage after mixing, thereby demineralizing the tooth surface.^4,10^ Further, as the chemical reaction proceeds, the constituent hydrophilic and acidic monomers are gradually consumed, and the cement becomes more hydrophobic and then finally polymerizes. Sufficient polymerization of the composite cement ensures minimal moisture absorption, hygroscopic expansion, and hydrolysis, which contributes to the long-term stability of the cement layer.^8,17^

A recently developed self-adhesive composite cement includes a self-adhesive composite-cement primer that provides a “touch-curing” function which enhances the polymerization reaction of cement.^13^ According to the manufacturer, the primer, which contains functional monomers, can improve the bond strength between the dentin and self-adhesive composite cement.^9^ In addition, when the cement is applied to the tooth surface after the self-adhesive composite-cement primer, polymerization of the cement layer is catalyzed immediately upon contact, ensuring sufficient polymerization. Therefore, applying the primer provided with the self-adhesive composite cement has been suggested as one of the methods for increasing the bond strength between the dentin and composite cement.^13^ Lower bond strength^21^ and lower degree of conversion^6^ are the two most important limitations of the self-adhesive composite cements compared to the multi-step composite cements.

The roughness of the dentin surface and the composition of the smear layer can be altered, depending on the type of rotary instrument used for tooth preparation.^15,22^ Several studies^7,12,14,22,23,25^ have reported that the smear layer of the dentin surface affects the penetration of the self-etching system in the tooth matrix. Penetration of functional monomers into the tooth surface is less on a rough dentin surface compared to on a smooth and thin smear layer.^5^

The wetness of the dentin surface also affects the penetration of the self-adhesive composite cement into the tooth matrix. Hydrated dentin substrates can promote the ionization of acidic monomers in the self-adhesive composite cement, but it has also been reported that excessive moisture can lessen the penetration of hydrophobic components in the cement.^10,14^ Thus, the exact effect of the characteristics of the dentin surface on bond strength between self-adhesive composite cement and dentin remains controversial.

The acidic functional monomers in the self-adhesive composite cement cannot completely dissolve the smear layer, and their penetration in the dentin surface is limited to the surface layer.^16^ Moreover, the application method of the composite cement and the characteristics of the dentin surface can affect the adhesive interface between the dentin and self-adhesive composite cement. Hence, different methods have been recommended for increasing the bond strength of the self-adhesive composite cement.^3,11,19^ However, there is insufficient information on the effect of the application method and the roughness or drying time of the dentin surface on the bond strength between composite cement and dentin, specifically in the case of composite cements that are applied with a recently released primer.

Therefore, this study investigated the effect of roughness and drying time of the dentin surface, as well as the number of coats of a self-adhesive composite-cement primer applied, on the bond strength of the composite cement and dentin. The null hypothesis of this study was that when the self-adhesive composite cement was applied to dentin, the roughness or drying time of the dentin surface, and the application method of a self-adhesive composite-cement primer would not affect the composite-cement bond strength.

## Materials and Methods

### Tooth Selection and Preparation of Specimens

**Fig 1:**
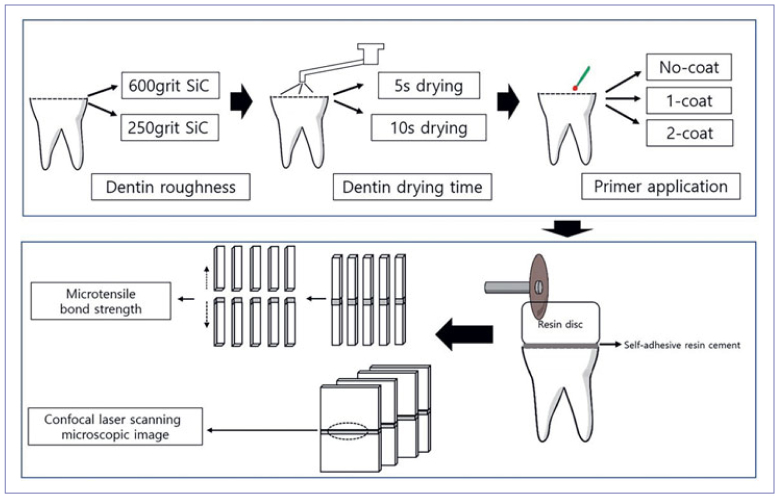
Schematic flow chart showing the preparation of specimens and experimental design.

Figure 1 shows the overall schematic workflow of the study. Sixty extracted caries-free human molars were used after approval from the Institutional Review Board of Pusan National University Dental Hospital (IRB, PNUDH-2020-037). The soft tissues of the external root surface were removed from the teeth. Next, they were disinfected using 0.5% chloramine solution and stored in distilled water at 4°C.

### Experimental Design and Preparation of Specimens

Table 1 lists the composition of the materials used in this study. The roots of the teeth used were embedded in self-curing acrylic resin (Tokuso Curefast, Tokuyama, Tokyo, Japan). The teeth were sectioned horizontally at the mid-coronal level using a water-cooled diamond saw (Accutom-50, Struers; Ballerup, Denmark) to obtain a flat, sound dentin surface.

**Table 1:** Composition of the materials used in this study

Material	Composition	Manufacturer
G-CEM one	Paste A: Fluoroaluminosilicate glass, UDMA, dimethacrylate, initiator, stabilizer, pigment, silicon dioxide, MDP Paste B: SiO_2_, trimethoxysilane, UDMA, 2-hydroxy-1,3-dimethacryloxypropane, MDP, 6-tert-butyl-2,4-xylenol, 2,6-di-tert-butyl-p-cresol, EDTA disodium salt dehydrate, vanadyl acetylacetonate, TPO, ascorbic acid, camphorquinone, MgO	GC; Tokyo, Japan
G-CEM one primer	Ethanol, MDP, 4-META, 2-hydroxy-1,3-dimethoxypropane, vanadyl acetylacetonate, 2,6-di-tert-butyl-p-cresol	GC
M1 GraceFil	Bis-GMA, UDMA, bis-EMA, zirconia, silica	GC

Bis-GMA: bisphenol A glycidyl methacrylate; UDMA: urethane dimethacrylate; bis-EMA: ethoxylated bisphenol A dimethacrylate; MDP: 10-methacryloyloxydecyl dihydrogenphosphate, 4-META: 4-methacryloxyethyl trimellitic anhydride; EDTA: ethylenediaminetetraacetate; TPO: 2,4,6-Trimethylbenzoyldiphenylphosphine oxide.

A total of 60 dentin specimens were cut perpendicular to the tooth axis and randomly divided into two groups to standardize the smear layer. The dentin surfaces of 30 teeth were polished with 250-grit silicon carbide abrasive paper for 60 s for the coarse roughness dentin group. For the fine roughness dentin group, the remaining 30 teeth were polished sequentially for 60 s with 250- and 600-grit silicon carbide abrasive papers. All specimens were polished using a polishing machine (Metaserv 250, Buehler; Lake Bluff, IL, USA) and then rinsed with water for 30 s. The remaining water was removed using an absorbent paper.

The two groups mentioned above were each divided into two further groups according to the drying time of the dentin surface: 5-s and 10-s drying. For the 10-s drying group, the dentin surface was dried for 10 s using a three-way air syringe with the air pressure adjusted to 1 bar using a pressure regulator, holding the air nozzle at 45 degrees to the surface at a distance of 1.5 cm. The 5-s drying group was obtained by blowing for 5 s using a three-way air syringe, with other parameters set as described above.

Polymerized composite resin blocks were made using a hybrid composite resin (M1 GraceFil A3 shade, GC; Tokyo, Japan) that was incrementally applied in 2-mm layers onto a silicone template (4 mm thickness and 9 mm diameter) and then light cured for 40 s using an LED light-curing unit (BluePhase G2, Ivoclar Vivadent; Schaan, Liechtenstein). The polymerized resin block was removed from the template and light cured on each side for 40 s. All polymerized composite resin blocks were polished with 250-grit silicon carbide abrasive paper using a polishing machine (Metaserv 250, Buehler) for 60 s, and then rinsed with water for 30 s.

Each group was then divided into three subgroups according to primer application: 1) in the no-coat (control) group, no primer was applied onto the dentin surface; 2) in the one-coat group (1-coat), one layer of the self-adhesive composite-cement primer (G-CEM one primer, GC) was applied onto the dentin surface for 10 s with a microbrush (Microbrush, Microbrush International; Grafton, WI, USA), and gently air blown for 5 s; 3) in the two-coat group (2-coat), two layers of the primer were applied onto the dentin surface, as described above.

After dentin surface pretreatment, the previously prepared polymerized composite resin blocks were bonded to the self-adhesive composite cement (G-CEM one, GC), followed by light curing of the four surfaces of the teeth for 10 s with an LED light-curing unit. The cemented specimens were stored in distilled water at room temperature for 24 h.

### Microtensile Bond Strength

The cemented specimens were sectioned into 1 mm x 1 mm x 10 mm sticks using a water-cooled diamond saw. Twelve specimens from each group were randomly selected and attached to a jig with cyanoacrylate cement (Zapit Dental Ventures of America; Corona, CA, USA). Subsequently, the microtensile strength (μTBS) of the specimens in each group was measured using a universal testing machine (Bisco; Schaumburg, IL, USA) at a crosshead speed of 1.0 mm/min until fracture. The μTBS values were calculated by dividing the load at failure by the cross-sectional bonding area.

### Failure Mode Analysis

After measuring the μTBS, all the debonded specimens were observed at a magnification of 40X under a microscope (Extaro 300, Carl Zeiss; Oberkochen, Germany) to determine the failure mode (each group n = 12). Failure modes were classified as follows: adhesive failure, which occurred at the interface between the dentin and cement or between the composite resin and cement; cohesive failure, which occurred within the cement layer; mixed failure, consisting of both cohesive and adhesive failures at the cement-dentin interface; and substrate failure, which occurred within the dentin or composite resin.

### Confocal Laser Scanning Microscopy

Twenty-four teeth sectioned horizontally at the mid-coronal level using a water-cooled diamond saw were prepared according to the dentin surface protocol described above to observe the bonding interface using CLSM. Rhodamine B fluorescent dye (Daejung, Seoul; Republic of Korea) was added to the primer of the self-adhesive composite cement, following which the primer was applied to the dentin. Fluorescein isothiocyanate (FITC) dye (Abbkine; Wuhan, Hubei, China) was added to the self-adhesive composite cement at a concentration of 0.01 wt%. All specimens were cut parallel to the tooth axis and polished. CLSM (CLSM; LSM-700, Carl Zeiss) was used to obtain images of the bonding interface in each group.^1^ Fluorescent images at 200X magnification were obtained and analyzed using the ZEN 2.6 (blue edition) software (Carl Zeiss).

### Statistical Analysis

Data were analyzed using three-way ANOVA and Tukey’s post-hoc comparisons test at a 95% confidence level. SPSS version 20 software (SPSS; Chicago, IL, USA) was used for statistical analysis.

## Results

Table 2 shows the results of the three-way ANOVA used for determining the effects of dentin roughness, dentin wetness, and primer application on μTBS between the composite cement and dentin. The results of this experiment showed that dentin roughness (F = 66.245, p = 0.000), drying time (F = 72.309, p = 0.000), and primer application (F = 73.511, p = 0.000) had significant effects on the bond strength between the cement and dentin, and these factors also showed significant interactions with each other (F = 6.11, p = 0.003).

**Table 2:** Results of 3-way ANOVA

Source	Sum of squares	df	Mean squares	F	p value
Dentin roughness	1073.11	1	1073.11	66.25	*0.000
Dentin wetness	1171.35	1	1171.35	72.31	*0.000
Primer application	2381.63	2	1190.82	73.51	*0.000
Dentin roughness x dentin wetness	56.63	1	56.63	3.50	0.064
Dentin roughness x primer application	51.24	2	25.62	1.58	0.210
Dentin wetness x primer application	46.60	2	23.30	1.44	0.241
Dentin roughness x dentin wetness x primer application	197.94	2	98.97	6.11	*0.003
Error	2138.29	132	16.20		

* Significant difference, p < 0.05.

**Fig 2:**
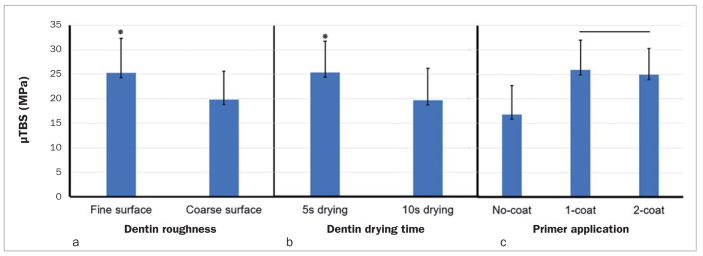
Mean values of the microtensile bond strength (MPa) for groups classified according to roughness and drying time of the dentin surface, and primer application. * Significant difference; horizontal bar (in primer application) indicates no significant difference.

Figure 2 shows the mean μTBS between the cement and dentin for each factor: roughness and drying time of the dentin surface and the application of primer. Regarding the roughness of the dentin surface, the fine roughness group (mean μTBS: 25.3 ± 7.1 MPa) showed significantly higher bond strength between cement and dentin than did the coarse roughness group (mean μTBS: 19.8 ± 5.8 MPa) (p = 0.000). Regarding the drying time of the dentin surface, the 5-s drying group (mean μTBS: 25.4 ± 6.4 MPa) showed a significantly higher bond strength than did the 10-s drying group (mean μTBS: 19.7 ± 6.5 MPa) (p = 0.000). For primer application, there was no significant difference between the 1-coat group (mean μTBS: 25.9 ± 6.1 MPa) and the 2-coat group (mean μTBS: 24.9 ± 5.4 MPa) (p = 0.692), and both groups showed significantly higher bond strength between the cement and dentin compared to the no-coat group (mean μTBS: 16.8 ± 5.9 MPa) (p = 0.000).

Table 3 shows results of the Tukey’s post-hoc test for all groups analyzed according to primer application and the dentin surface condition. The highest μTBS (31.8 ± 3.1 MPa) was seen in the 1-coat/fine roughness/5-s drying group, and the lowest μTBS (13.4 ± 2.7 MPa) was found in the no-coat/coarse roughness/10-s drying group.

**Table 3:** Mean ± standard deviation of microtensile bond strength (MPa) between dentin and cement for all experimental groups

Primer application	Dentin roughness	Dentin wetness	Mean ± SD
No-coat	Fine	Dry	13.4 ± 3.2^a^
		Moist	24.6 ± 5.4^def^
	Coarse	Dry	13.4 ± 2.7^a^
		Moist	16.0 ± 3.4^ab^
1-coat	Fine	Dry	27.2 ± 4.0^efg^
		Moist	31.8 ± 3.1^ g^
	Coarse	Dry	18.7 ± 3.8^abc^
		Moist	26.0 ± 4.6^def^
2-coat	Fine	Dry	24.9 ± 4.4^def^
		Moist	29.9 ± 4.2^fg^
	Coarse	Dry	20.8 ± 5.2^bcd^
		Moist	24.1 ± 3.6^cde^

Different superscript letters indicate statistically significant differences according to Tukey’s post-hoc test (p < 0.05).

**Fig 3:**
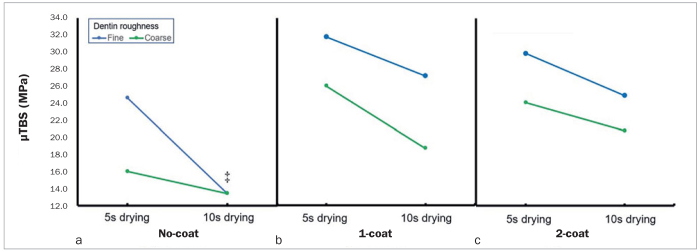
Mean values of the microtensile bond strength (MPa) for groups classified according to roughness and drying time of the dentin surface, and the number of primer applications. ‡ no significant difference.

Figure 3 shows the pairwise comparison of the bond strength between the cement and dentin with respect to dentin roughness and drying time according to the number of primer applications. In all groups, the bond strength of the 10-s drying group was lower than that of the 5-s drying group, regardless of the roughness of the dentin. Regarding the roughness of the dentin surface, the bond strength of the fine roughness group was significantly higher than that of the coarse roughness group, except for the 10-s drying group in the no-coat group (p < 0.05).

**Fig 4:**
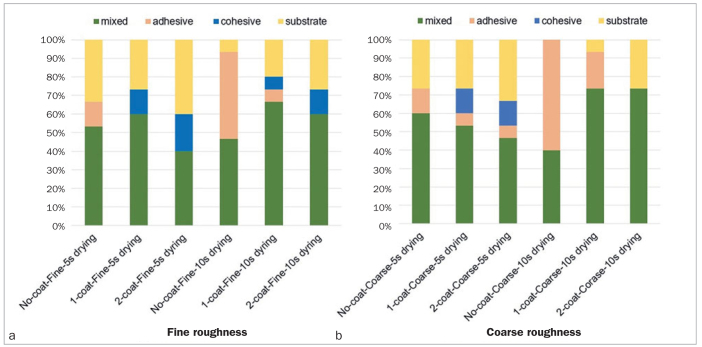
Failure modes for different groups.

Figure 4 shows the fracture patterns of the fractured specimens. Mixed fractures were common in most groups. Regardless of the dentin surface condition, adhesive failure was predominant in the no-coat group, whereas in the no-coat/coarse/10-s drying group, adhesive failure was observed in 60% of the debonded specimens. Cohesive and substrate failure patterns were observed more frequently in the fine roughness groups than in the coarse roughness groups. In particular, in the 2-coat/fine roughness/5-s drying group, cohesive failure was observed in 20% of debonded specimens and substrate failure was seen in 40%.

**Fig 5:**
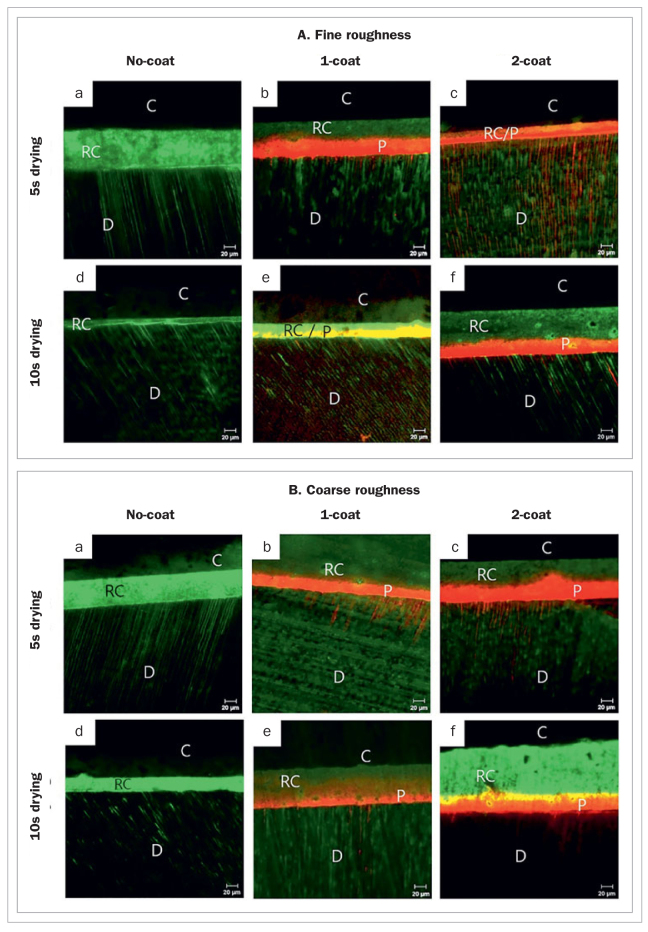
Confocal microscopy images showing the bonding interface between the composite block (C) and dentin (D) using G-Cem One self-adhesive composite cement (RC). The self-adhesive composite cement (RC) is labeled with fluorescein and the self-adhesive composite-cement primer (P) is stained with rhodamin B, showing green and red fluorescent colors, respectively. In the no-coat groups, the self-adhesive composite cement poorly penetrates the dentin (Figs 5A-a, 5A-d, 5B-a,and 5B-d). In the primer coated groups, the penetration of the self-adhesive composite cement in the dentin surface is higher than that in the no-coat groups. In the 2-coat/fine roughness/5-s drying dentin group (Fig 5A-c), the primer sufficiently penetrates the dentin compared to the other groups.

Figure 5 shows the CLSM images for each group. In the no-coat group, it was observed that the self-adhesive composite cement poorly penetrated the dentin. In particular, the 10-s drying group showed a worse penetration pattern than did the 5-s drying dentin group (Figs 5A-d and B-d). In the primer-coated groups, the primer was observed to be in close contact with the dentin surface. Moreover, the penetration of the self-adhesive composite cement into the dentin surface was higher than in the no-coat groups. In the 2-coat/fine roughness/5-s drying dentin group (Figs 5A-c), the primer sufficiently penetrated the dentin compared to the other groups. In the primer coating groups, the coarse roughness vs the fine roughness dentin group, and the 10-s vs the 5-s drying group showed insufficient penetration of the self-adhesive composite cement in the dentin.

## Discussion

This study investigated the effects of roughness of the dentin surface after it was cut, the drying time of the dentin surface before a bonding procedure, and the application of the self-adhesive composite cement with primer on the adhesive interface between the cement and dentin on the bond strength of the composite cement.

Penetration of the composite cement into the dentin surface depends on cement viscosity, contact angle between the two surfaces, roughness of the dentin surface, and the cutting grit size of the rotary instrument.^15,24^ A smear layer of approximately 0.9–3.5 µm thickness is created when teeth are cut using a rotating dental instrument for restorative treatment.^12^ This smear layer is composed of collagen debris and mineral particles and is loosely attached to the tooth surface, interfering with effective adhesion. Several studies have reported that the thickness, roughness, and density of the smear layer are affected by the type of rotary dental instruments used during cavity preparation.^7,16,22^ Oliveira et al^22^ reported that the 240-grit abrasive paper produced a coarser, thicker smear layer than the 600-grit abrasive paper. Further, the characteristics of the smear layer can interfere with open tubules on the dentin surface when a self-etching primer comprising functional monomers is applied. The rougher the dentin surface is, the thicker the smear layer, which may adversely affect the penetration of the functional monomers. This indicates that a rough dentin surface may be disadvantageous in forming chemical bonds between the functional monomers of the self-adhesive composite cement. In our experiments, when the self-adhesive composite cement was applied, the bond strength between cement and dentin on the coarse dentin surface (formed with the 240-grit abrasive paper) was significantly lower than that for the fine dentin surface (formed with the 600-grit abrasive paper) (p = 0.000). The CLSM images (Fig 5) showed that the penetration of the self-adhesive composite cement into dentin was insufficient in the coarse roughness group vs the fine roughness group.

Moisture on the surface of the dentin promotes the ionization of functional monomers in the self-adhesive composite cement. In the presence of moisture, the functional monomer is ionized and spontaneously etches the dentin surface.^10^ Several studies have suggested that the moisture level of the dentin surface might play an important role in the strength of the bond between the self-adhesive composite cement and teeth.^2,10,14,18^ In the present study, the bond strength between cement and dentin of the 5-s drying group was significantly higher than that of the 10-s drying group (p = 0.000). This indicates that the moisture on the dentin surface activates the functional monomers in the cement and improves their penetration into the dentin surface.

On the other hand, micromechanical retention and chemical interactions between functional monomers of the self-adhesive composite cement and the hydroxyapatite of teeth occur only superficially on dentin.^19,20^ To improve the cement’s infiltration, several methods, such as pretreatments on the dentin surface before application of cement, have been tested.^3,11,19,25^ Recently, the application of a self-adhesive composite-cement primer containing functional monomers such as 10-MDP and 4-META and a polymerization promoting component on the dentin surface before the application of the self-adhesive composite cement has been recommended.^13^ According to the manufacturer’s description, this should increase the penetration of the composite cement in the dentin and activate the polymerization of the cement layer.^9^ In this study, the primer-coated group had significantly higher bond strengths than the no-coat group (p = 0.000). The CLSM images showed that the self-adhesive composite cement poorly penetrated the dentin in the no-coat groups. Alternatively, in the primer-coated groups, the primer was observed to be in close contact with the dentin surface; moreover, there was greater penetration of the self-adhesive composite cement into the dentin surface, compared to that in the no-coat group. This result indicated that the application of the primer improved the fluidity and wettability of the cement on the dentin surface. Among the primer-coated groups, CLSM showed that the 2-coat/fine roughness/5-s drying group had the best penetration into the dentin compared to the other groups (Fig 5 A-c). Moreover, in the primer-coated groups, there was less penetration of the self-adhesive composite cement into the dentin in the coarse-roughness group compared to the fine-roughness group and in the 1-s drying group compared to the 5-s drying group. This was consistent with the bond strength results between the composite cement and dentin. This indicates that the fine-roughness and 5-s drying conditions improve the bond strength of the self-adhesive composite cement.

In this study, the no-coat group showed lower bond strength in the 10-s drying group, regardless of the roughness of the dentin (Fig 3). In the primer-coated groups, the rougher the dentin surface, the lower the bond strength, regardless of the drying time of the dentin surface. These results indicate that the primer makes the dentin sufficiently moist, and, as a result, the influence of the drying time of the dentin surface on bond strength is minimized. When applying the self-adhesive composite cement, primer application can compensate for the influence of the dentin-surface drying time by improving it. Therefore, in the primer-coated groups, the results were mainly affected by the roughness of the dentin surface. Based on these results, the null hypothesis was rejected.

This study had some limitations. The experiments were performed without reproduction of dentinal fluid under pulpal pressure in the extracted teeth, and long-term bond strength between the composite cement and dentin was not assessed. Therefore, when using the self-adhesive composite cement for bonding indirect restorations, a self-adhesive composite-cement primer can be used to improve the bond strength between the composite cement and dentin, while considering the drying time and roughness of the dentin.

## Conclusion

In the present study, the application of a self-adhesive composite-cement primer prior to self-adhesive composite-cement significantly application improved the bond strength between the composite cement and dentin compared to the application of the cement alone. In addition, when the self-adhesive composite cement was applied, the roughness and drying time of dentin had a significant effect on the bond strength between the composite cement and dentin. The fine-roughness group showed higher bond strength than the coarse-roughness group, and the 5-s dentin drying time showed higher bond strength than the 10-s dentin drying time.

## Clinical relevance:

Use of a self-adhesive composite-cement primer with self-adhesive composite cement (G-CEM One) for bonding indirect restorations improves the bond strength between the composite cement and dentin. Additionally, the roughness and drying time of dentin have an effect on bond strength.
